# Determining the optimal duration of oral adjuvant chemotherapy in locoregionally advanced nasopharyngeal carcinoma

**DOI:** 10.1038/s41416-025-03033-1

**Published:** 2025-05-06

**Authors:** Hui Cheng, Jie Chen, Yifu Li, Yuchen Li, Chunfung TSE, Bowen Shen, Shibing Li, Qiuyan Chen, Linquan Tang, Haiqiang Mai, Liting Liu

**Affiliations:** 1 State Key Laboratory of Oncology in South China, Collaborative Innovation Center for Cancer Medicine, Guangdong Key Laboratory of Nasopharyngeal Carcinoma Diagnosis and Therapy, Guangdong Provincial Clinical Research Center for Cancer, Sun Yat-sen University Cancer Center, Guangzhou, PR China; 2 Department of Nasopharyngeal Carcinoma, Sun Yat-sen University Cancer Center, Guangzhou, PR China; 3 Department of Clinical Laboratory, Biomedical Innovation Center, The Sixth Affiliated Hospital, Sun Yat-sen University, Guangzhou, PR China

**Keywords:** Head and neck cancer, Diseases

## Abstract

**Background:**

Concurrent chemoradiotherapy (CCRT) followed by adjuvant chemotherapy (AC) is the standard treatment for locoregionally advanced nasopharyngeal carcinoma (LA-NPC). However, the optimal duration of oral AC remains poorly defined.

**Methods:**

This study examined newly diagnosed patients between April 2017 and December 2020. The primary endpoint was overall survival (OS). Restricted cubic splines (RCS) and Kaplan–Meier method were used to evaluate the relationship between AC maintenance and survival. Inverse probability of treatment weighting (IPTW) was used to control for confounding factors.

**Results:**

The RCS demonstrated an L-shaped association between oral AC maintenance and OS. The risk of mortality was relatively flat after 12 months. Patients with oral AC duration >186 days (defined by RCS) had a significantly better OS (HR 0.23 [95% CI 0.10–0.55], log-rank p < 0.001), with a higher 3-year OS rate (98.7% [95% CI 96.8–100.0] vs 88.3% [95% CI 82.5–94.5]). For patients with pretreatment Epstein-Barr virus (EBV) DNA level >4000 copies/mL, mortality risk decreased to 1 at 194 days of AC duration.

**Conclusions:**

The optimal duration of oral AC after CCRT was >186 days (6 months) for LA-NPC. And the maintenance beyond 12 months may not bring additional benefits.

## Introduction

Nasopharyngeal carcinoma (NPC) is a malignant tumour originating from the epithelial cells lining the nasopharynx [1]. More than 70% of NPC patients are diagnosed with locoregionally advanced disease [2]. Concurrent chemoradiotherapy (CCRT) with platinum agents is the mainstay treatment [3]. However, although most patients achieve complete clinical remission after CCRT, ~20–30% of patients experience disease progression within 3 years [4].

The landmark Intergroup 0099 trial was the first to report a significant therapeutic benefit of cisplatin-based CCRT plus cisplatin–fluorouracil adjuvant chemotherapy (AC) over radiotherapy alone [5]. Subsequent trials in endemic populations have achieved consistent results, establishing CCRT followed by AC as the standard treatment for locoregionally advanced nasopharyngeal carcinoma (LA-NPC) [6–8].

However, the main limitation of intravenous AC is its poor tolerability. Previous trials have reported that only 50–60% of patients could complete CCRT and intravenous AC treatment [9–11]. Oral administration is a promising treatment method, with good compliance and low toxicity. Adjuvant capecitabine and S-1 are effective when added to CCRT in patients with LA-NPC [12, 13]. These two oral drugs are preferentially converted to metabolically active fluorouracil in vivo and have the potential to replace traditional intravenous fluorouracil. Studies and systemic reviews from several malignancies confirmed their comparable efficacy [14–17].

Based on large clinical trials, the typical duration for oral maintenance therapy ranges from 3 months to 2 years [18, 19] and is influenced by the clinician’s subjective preferences. Several systematic reviews have explored the correlation between chemotherapy duration and survival outcomes in other malignancies [20–22]. However, the optimal duration of oral AC after CCRT remains poorly defined in patients with LA-NPC. In addition, plasma Epstein-Barr virus (EBV) is a reliable prognostic biomarker for NPC [23, 24]. Patients with differing EBV DNA levels exhibit varying risks of treatment failure, and it remains uncertain whether a standardised treatment duration should be administered to patients with different EBV DNA levels.

Therefore, this study aimed to examine the association between oral AC duration and overall survival, with the hypothesis that an optimal threshold could be determined.

## Materials and methods

### Study population

**Fig. 1:**
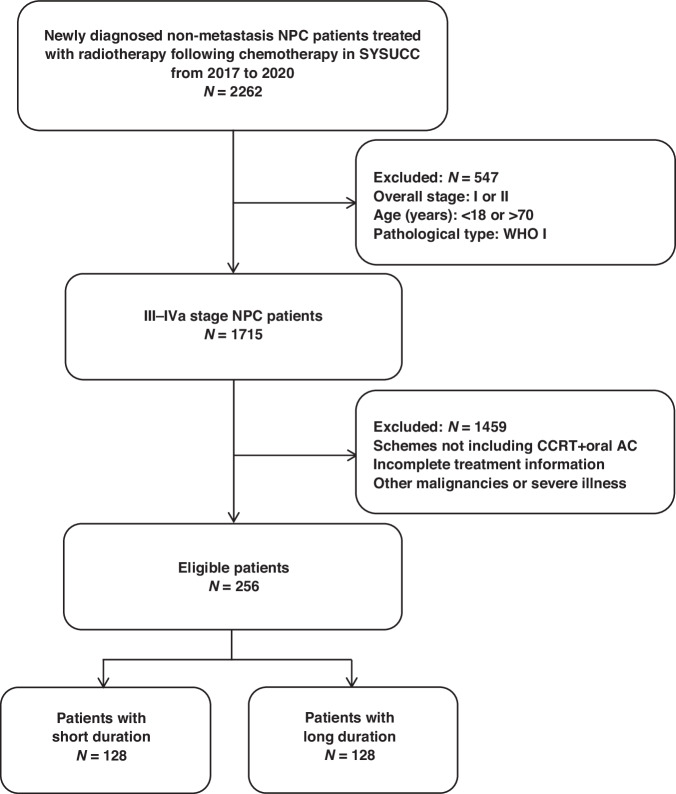
Flow chart of the patient selection process. NPC nasopharyngeal carcinoma, SYSUCC Sun Yat-sen University Cancer Center, WHO World Health Organization, CCRT concurrent chemoradiotherapy, AC adjuvant chemotherapy.

Between April 2017 and December 2020, data on newly diagnosed patients were sourced from the Single Disease Database of the Sun Yat-sen University Cancer Center. The inclusion criteria were as follows: Patients with histologically confirmed non-keratinising NPC (World Health Organization types II and III); American Joint Committee on Cancer staging system (eighth edition) staged III-IVa; those who had undergone platinum-based CCRT followed by oral AC; age 18–70 years; Eastern Cooperative Oncology Group performance status score of 0–1; satisfactory liver, kidney, and bone marrow function; availability of complete treatment information and pretreatment plasma EBV DNA results; and no history of other malignancies. The key exclusion criteria were metastasis or recurrence of NPC at the time of diagnosis, lactation, pregnancy, insufficient clinical data, or severe coexisting illnesses. To minimise the influence of outliers, patients within the top and bottom 2% of the AC duration were excluded [25], resulting in the final distribution illustrated in Fig. 1.

The study protocol was approved by the Research Ethics Committee of the Cancer Center of Sun Yat-sen University (Guangdong, China). Written or oral informed consent was obtained from all participants.

Pretreatment evaluation, including complete patient history, physical examination, haematology and biochemistry profiles, magnetic resonance imaging (MRI) of the nasopharynx and neck, whole-body computed tomography (CT), chest radiography, abdominal sonography, bone scintigraphy or whole-body ^18^F-positron emission tomography-computed tomography were performed. Pretreatment plasma EBV DNA levels were evaluated using quantitative reverse transcription polymerase chain reaction assay for subgroup analysis [26].

### Treatment

All patients received oral AC after CCRT. The patients underwent intensity-modulated radiotherapy, with the primary tumour and enlarged lymph nodes included in the gross tumour volume, adhering to the institutional guidelines and principles [27]. Five daily fractions per week were prescribed with an accumulated dose of 68–70 Gy. Concurrent platinum-based chemotherapy (100 mg/m^2^ every 3 weeks or 40 mg/m^2^ every week) was administered simultaneously with radiotherapy.

The oral AC regimen included capecitabine (1000 mg/m^2^ BID, days 1–14) and S-1 (40–60 mg BID, days 1–14). The dose of S-1 was determined by the body surface area (BSA): 40 mg twice a day for BSA < 1.25 m^2^; 50 mg twice a day for 1.25 m^2^ ≤ BSA < 1.5 m^2^; and 60 mg twice a day for BSA ≥ 1.5 m^2^. Chemotherapy dose adjustments were permitted for adverse events (Common Terminology Criteria for Adverse Events, version 5.0), but AC would not be initiated until the adverse events recover to grade <2.

In total, 155 participants (60.5%) underwent induction chemotherapy (IC). The IC regimens were listed as follows: TPF (docetaxel [60 mg/m^2^, day 1], cisplatin [60 mg/m^2^, day 1], and 5-fluorouracil [3 g/m^2^, continuous intravenous infusion for 120 h]), PF (cisplatin [75 mg/m^2^, day 1], 5-fluorouracil [4 g/m^2^]), TP (docetaxel [75 mg/m^2^, day 1] or paclitaxel [135 mg/m^2^, day 1] and cisplatin [75 mg/m^2^, day 1]), GP (gemcitabine [1.0 g/m^2^, days 1 and 8], cisplatin [80 mg/m^2^, day 1]) for two to three cycles.

### Follow-up and outcomes

Patients were followed up every 3 months in the first 3 years and then every 6 months until December 31, 2023. Haematological and biochemical profiles, MRI of the head and neck, chest scans (radiography or CT), abdominal scans (sonography or CT), and optic nasopharyngoscopy were performed annually or upon clinical indication of tumour progression. Further examinations were performed as clinically indicated.

The primary endpoint was overall survival (OS), defined as the time interval between the initial date of diagnosis and death from any cause. The secondary endpoints were progression-free survival (PFS; interval from the first date of diagnosis to disease progression or death from any cause), locoregional recurrence-free survival (LRRFS), and distant metastasis-free survival (DMFS), which corresponded to the time interval between the first locoregional recurrence and distant metastasis or death from any cause.

### Statistical analysis

A multivariable Cox proportional hazards model with restricted cubic splines (RCS) was constructed to analyze the association between oral AC duration and OS. The RCS is a smooth junction of polynomial functions under the assumption of a nonlinear relationship [28]. Given the outstanding flexibility for fitting risk function changes and adjustment for confounding factors, the RCS has been widely applied to survival analysis [29–31]. A spline with three knots was defined [32]. The following clinical characteristics were included in the multivariate analysis: sex, age (median age 46 years, interquartile range [IQR] 37–54), family history of NPC, tumour stages (T and N categories separately, and overall stage), schemes (with or without induction chemotherapy), and pretreatment plasma EBV DNA levels (categorised as ≤4000 copies/mL and >4000 copies/mL, as previously described [27]). The cohort was then divided into short and long duration groups based on the cutoff value defined by the RCS curve.

The chi-square test was used to compare the distribution of clinical factors between the two groups. To minimise the bias arising from clinical characteristics, we used inverse probability of treatment weighting (IPTW) in our study [33]. When the standardised mean differences (SMDs) were 0.1 or less after IPTW, the covariate was considered to have no between-group difference [34]. Survival curves and outcomes were analyzed using the Kaplan–Meier method and log-rank test. Hazard ratios (HRs) were calculated by univariate Cox regression analysis. A multivariable Cox proportional hazards model was used to evaluate the independence of the prognostic values. Two-sided *P* < 0.05 indicated statistical significance. All statistical analyses were performed using SPSS 27.0 (IBM Corp., Armonk, NY, USA) and R software 4.3.2 (R Core Team, Vienna, Austria), R packages: rms, version 6.7-1; plotRCS, version 0.1.5; survival, version 3.5-7; and survminer, version 0.4.9. The codes used and/or analysed during the current study available from the corresponding author on reasonable request.

## Results

### Patient characteristics of the total cohort

This study included 256 patients with AC duration between 21 and 1588 days (median age: 46 years (IQR 37–54) and 169 (66.0%) participants were men). 127 (49.6%) patients received capecitabine and 129 (50.4%) received S-1. The median concurrent cisplatin dose was 200 mg/m^2^ (IQR 200–240) [35, 36]. After a median follow-up period of 50 months (IQR 37–62), disease progression and mortality were reported in 70 (27.3%) and 27 (10.5%) patients, respectively. The distribution of failure sites included 21 locoregional recurrences, 39 distant metastases, and 10 combined cases. The median duration of oral AC maintenance was 187 days (IQR 100–368).

### RCS to identify the association between oral adjuvant maintenance and OS

**Fig. 2:**
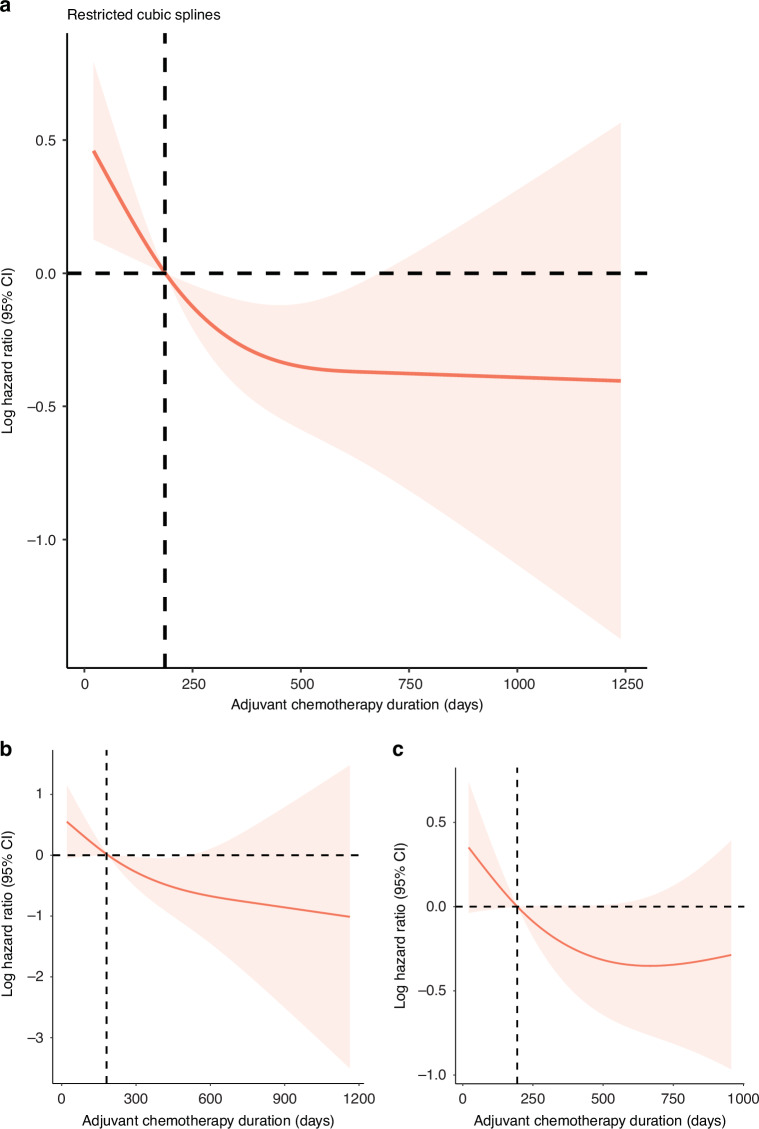
Restricted cubic spline modelling of the relationship between adjuvant chemotherapy duration and mortality risk. The hazard ratios (HR) derived from the multivariate Cox model, and it decreased to 1 at 186 days (**a**). For low- (**b**) and high-risk (**c**) subgroups defined by pretreatment EBV DNA level, The HRs decreased to 1 on days 181 and 194. The shaded areas represent the 95% confidence intervals (CIs) of the adjusted HRs.

A multivariable-adjusted Cox hazard model with RCS demonstrated an L-shaped association between oral AC maintenance and OS (total *p* = 0.03) (Fig. 2a). The risk of mortality decreased rapidly as the duration extended, and it was relatively flat after 12 months. The hazard ratio decreased to 1 at 186 days. In patients with treatment duration ≤186 days, each additional week of maintenance was associated with 0.42 months of increase in OS (95% CI 0.02–0.81, *p* = 0.04).

Based on the pretreatment EBV DNA value of 4000 copies/mL, the patients were further divided into low- and high-risk subgroups, and RCS models were constructed. Patients in the low-risk group (*n* = 157, 61.3%) exhibited a marked decrease in mortality risk with an increase in AC duration, which was attenuated after approximately 15 months (Fig. 2b). The HR decreased to 1 at 181 days. Additionally, AC maintenance was also negatively correlated with mortality risk in the high-risk group (*n* = 99, 38.7%), with the HR decreased to 1 at 194 days. However, a slight increase was observed after 22 months (Fig. 2c).

### Comparison of baseline characteristics between patients with short and long durations

Based on the cutoff value of 186 days defined by the RCS curve, patients were assigned to the short (≤186 days) and long duration groups (>186 days) (*n* = 128 in each group), respectively. The median completed course was 4.4 (IQR 2.0–7.5) in the short duration group and 17.4 (IQR 11.9–25.2) in the long group. Compared with the short duration group, the proportion of patients with stage T1-2 (*p* = 0.03), IVa disease (*p* = 0.02) and receiving IC (p < 0.001) in the long duration group were higher. However, after IPTW adjustment, all listed cofounders were well-balanced between groups (Table 1).

**Table 1:** Baseline characteristics of the short and long duration groups before and after weighting.

Characteristic	Unweighted, N%			Weighted, N%			
	Short duration	Long duration	*p* value	Short duration	Long duration	*p* value	SMD
	*n* = 128	*n* = 128					
Sex			0.69			0.82	0.03
Female	42(33)	45(35)		(31)	(33)		
Male	86(67)	83(65)		(69)	(67)		
Age			0.06			0.96	0.007
<45 years	53(41)	68(53)		(46)	(46)		
≥45 years	75(59)	60(47)		(54)	(54)		
Family history of NPC			0.18			0.89	0.02
No	120(94)	114(89)		(91)	(91)		
Yes	8(6)	14(11)		(9)	(9)		
T category^a^			0.03			0.96	0.008
T1-2	5(4)	14(11)		(8)	(8)		
T3-4	123(96)	114(89)		(92)	(92)		
N category^a^			0.90			0.95	0.008
N0-1	46(36)	45(35)		(35)	(35)		
N2-3	82(64)	83(65)		(65)	(65)		
Overall stage^a^			0.02			0.89	0.02
III	60(47)	41(32)		(40)	(39)		
IVa	68(53)	87(68)		(60)	(61)		
Schemes			<0.001			0.96	0.007
CCRT + AC	67(52)	34(27)		(40)	(40)		
IC + CCRT + AC	61(48)	94(73)		(60)	(60)		
Pretreatment EBV DNA			0.90			0.85	0.03
≤4000 copies/mL	79(62)	78(61)		(61)	(63)		
>4000 copies/mL	49(38)	50(39)		(39)	(37)		

The chi-squared test was used to calculate *p* value. All variables were transformed into categorical variables.

*SMD* standardised mean difference, *NPC* nasopharyngeal carcinoma, *CCRT* concurrent chemoradiotherapy, *AC* adjuvant chemotherapy, *IC* induction chemotherapy, *EBV* Epstein–Barr virus.

^a^According to the eighth edition of UICC/AJCC staging system.

### Prognostic value of oral AC maintenance time

**Fig. 3:**
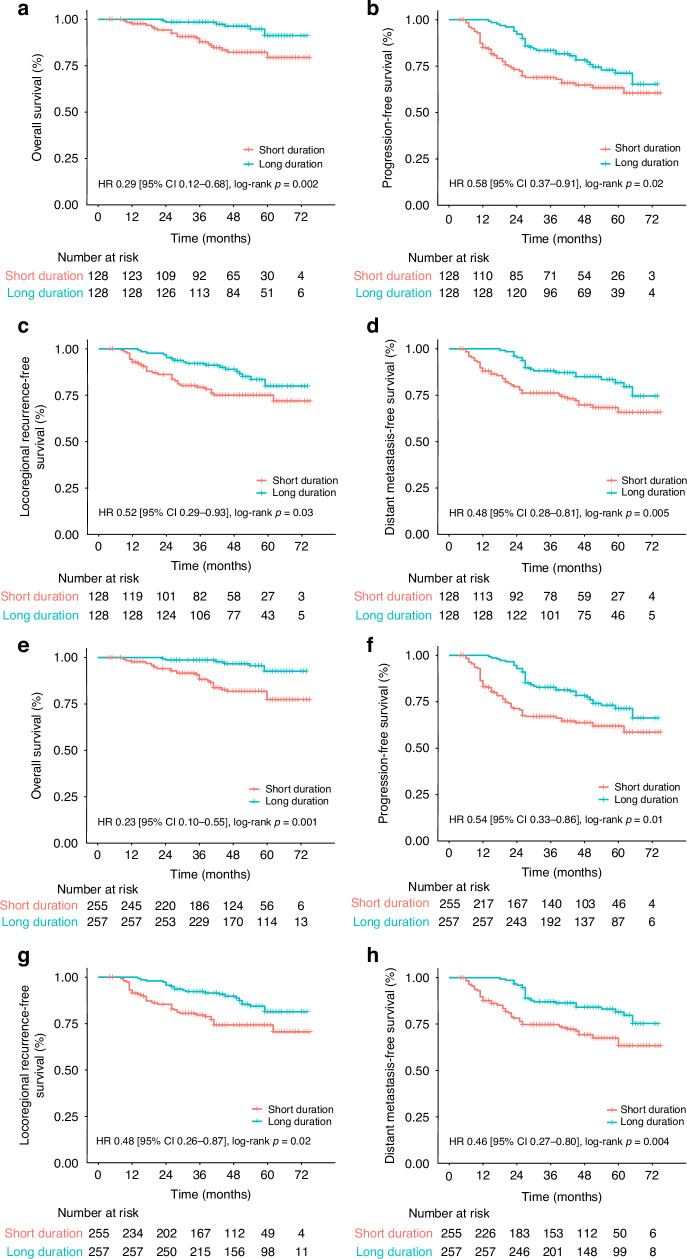
Kaplan-Meier curves of survival outcomes. Kaplan–Meier curves of OS (**a**), PFS (**b**), LRRFS (**c**), and DMFS (**d**). The inverse probability of treatment weighting-adjusted Kaplan–Meier curves of OS (**e**), PFS (**f**), LRRFS (**g**), and DMFS (**h**). Univariate Cox proportional hazards model (**a**–**h**) was used to calculate HRs and 95% CIs. HR hazard ratio, CI confidence interval, OS overall survival, PFS progression-free survival, LRRFS locoregional recurrence-free survival, DMFS distant metastasis-free survival.

At the time of analysis, the IPTW-adjusted Kaplan–Meier curves illustrated that OS at 3 years was 88.3% (95% CI 82.5–94.5) in the short duration group, compared with 98.7% (95% CI 96.8–100.0) in the long duration group (HR 0.23 [95% CI 0.10–0.55], log-rank p < 0.001), which indicated that patients with longer AC maintenance had a lower mortality rate. Furthermore, patients in the long duration group had a significantly higher 3-year PFS rate (67.1% vs. 82.8%, HR 0.54 [95% CI 0.33–0.86], log-rank *p* = 0.01), LRRFS rate (79.7% vs. 92.3%, HR 0.48 [95% CI 0.26–0.87], log-rank *p* = 0.02), and DMFS rate (74.7% vs. 87.0%, HR 0.46 [95% CI 0.27–0.80], log-rank *p* = 0.006) (Fig. 3).

After weighting, the maintenance group had an independent effect on OS (HR 0.22 [95% CI 0.09–0.51], *p* < 0.001), PFS (HR 0.51 [95% CI 0.32–0.83], *p* = 0.006), LRRFS (HR 0.47 [95% CI 0.26–0.86], *p* = 0.01), and also DMFS (HR 0.41 [95% CI 0.23–0.73], *p* = 0.002). In addition, the pretreatment plasma EBV DNA level (HR 2.24 [95% CI 1.01–4.98], *p* = 0.047) was also independent for OS (Table 2). Detailed information on the multivariate analysis of PFS, LRRFS, DMFS and univariate analysis of OS was summarised in eTable 1, 2.

**Table 2:** Multivariate Cox regression analysis of OS before and after weighting.

Characteristic	Unweighted		Weighted	
	HR (95%CI)	*p* value	HR (95%CI)	*p* value
Sex	1.11 (0.49–2.52)	0.80	1.05 (0.44–2.49)	0.91
Female	Reference			
Male				
Age (years)	1.56 (0.71–3.44)	0.26	1.40 (0.65–3.04)	0.39
<45	Reference			
≥45				
Family history of NPC	1.26 (0.28–5.57)	0.76	2.17 (0.65–7.23)	0.21
None	Reference			
Yes				
T category^a^	1.64 (0.36–7.49)	0.53	2.87 (0.48–17.23)	0.25
T1-2	Reference			
T3-4				
N category^a^	1.74 (0.67–4.48)	0.25	2.17 (0.82–5.72)	0.12
N0-1	Reference			
N2-3				
Overall stage^a^	1.78 (0.72–4.40)	0.21	2.18 (0.88–5.40)	0.09
III	Reference			
IVa				
Schemes	1.36 (0.59–3.16)	0.47	1.72 (0.70–4.24)	0.23
CCRT + AC	Reference			
IC + CCRT + AC				
Pretreatment EBV DNA (copies/mL)	2.44 (1.06–5.60)	0.04	2.24 (1.01–4.98)	0.047
≤4000	Reference			
>4000				
Duration group	0.24 (0.10–0.61)	0.002	0.22 (0.09–0.51)	<0.001
Short duration	Reference			
Long duration				

A Cox proportional hazards regression model was used to perform multivariate analysis. HRs and their 95% CIs were calculated for Sex (Male vs. Female); Age in years (≥45 vs. 4000 vs. ≤4000); Duration group (Long duration vs. Short duration).

*HR* hazard ratio, *CI* confidence interval, *NPC* nasopharyngeal carcinoma, *CCRT* concurrent chemoradiotherapy, *AC* adjuvant chemotherapy, *IC* induction chemotherapy, *EBV* Epstein–Barr virus.

^a^According to the eighth edition of UICC/AJCC staging system.

A Cox proportional hazards regression model was used to perform multivariate analysis. HRs and their 95% CIs were calculated for Sex (Male vs. Female); Age in years (≥45 vs. <45); Family history of NPC (Yes vs. No); T category (3–4 vs. 1–2); N category (2–3 vs. 0–1); Overall stage (IVa vs. III); Schemes (IC + CCRT + AC vs. CCRT + AC); Pretreatment EBV DNA (copies/mL) (>4000 vs. ≤4000); Duration group (Long duration vs. Short duration).

## Discussion

In this study, we explore the association between AC duration and prognosis in patients with LA-NPC. The optimal durations of oral AC was >186 days (6 months). The maintenance beyond 12 months may not bring additional benefits. For patients with pretreatment EBV DNA > 4000 copies/mL, the maintenance should be extended to 194 days.

The Intergroup 0099 trial has enhanced our understanding of AC. A 2016 network meta-analysis that assessed the seven most common treatments for LA-NPC reemphasised that CCRT + AC achieved the highest benefit and consistent improvement in all survival outcomes [37]. Although Chen et al. previously reported that intravenous AC with cisplatin-fluorouracil did not improve failure-free survival [9], they recently suggested that the addition of metronomic oral capecitabine after CCRT achieved remarkable survival outcomes and a manageable safety profile [12]. In addition to demonstrating adjuvant efficacy, this study explored the potential of oral AC, which is consistent with the findings of two other studies on the use of oral adjuvant capecitabine and S-1 in patients with high-risk factors [13, 18]. The latest meta-analysis concluded that AC showed the greatest impact on locoregional progression, while IC appeared to be the most effective against distant metastasis [38]. As there was insufficient evidence from a head-to-head comparison between IC and AC, it remains unclear which treatment is superior. The preliminary results of our studies (NCT03306121), directly compared IC + CCRT with CCRT + AC based on PF regimen, and found no significant difference in survival between the two groups. The National Comprehensive Cancer Network (NCCN) guidelines (version 2.2024) recommended CCRT + AC for the treatment of LA-NPC with level IIA evidence. However, different maintenance durations for oral AC were reported in existing literature and the optimal duration time remains unclear. Zong et al. revealed that patients with stage N3 may benefit from ≥12 cycles of S-1 [19]. In contrast, Dong et al. retrospectively reported superior survival outcomes in metronomic AC > 3 months based on larger sample size, however, the patient cohort was selected between 2013 and 2020, with a median follow-up period of 4 years. The wide span of patient enrolment, the variable length of follow-up period, and the improvement of NPC treatment patterns, may affect the results [39]. In the field of breast cancer, a systematic review also concluded that a longer first-line chemotherapy duration was associated with better OS and PFS [20]. Furthermore, the metronomic dosing regimen of oral AC has recently drawn considerable attention. Studies have reported on its potential to modulate immune responses, particularly by enhancing T-cell activity and reducing the activity of immune suppressors [40–43]. Nevertheless, the superiority of this dosing strategy over conventional full dose chemotherapy remains to be substantiated by further evidence.

We constructed an adjusted RCS model to examine the relationship between oral AC duration and OS, which demonstrated an L-shaped association. The risk of mortality decreased rapidly as the duration increased, and the HR decreased to 1 at 186 days, with a plateau after approximately 12 months. This finding supports the hypothesis that AC offers maximal benefit within a certain time frame and may not provide additional benefit beyond a specific threshold. To further explore the relationship between oral AC maintenance and survival, the participants were divided into short and long duration groups based on the cutoff duration of 186 days. After IPTW adjustment, the differences between the two groups were minimised, and duration group was still an independent prognostic factor for all survival endpoints. IC scheme was not significant in the multivariate analysis, which was consistent with Chen’ conclusion, AC achieved a survival benefit regardless of the use of IC [12]. In patients who received IC and those who did not, there was a similar trend of L-shaped association between oral AC maintenance and OS. However, our no IC cohort has a much smaller number of patients, the results need further confirmation (eFigure 1, 2, eTable 3–6). Regarding the IPTW-adjusted survival outcomes, patients who received prolonged oral AC maintenance >186 days had significantly better OS, PFS, LRRFS, and DMFS rate than those who received duration below this threshold. AC eradicates both residual disease at locoregional sites and subclinical micro-metastasis [44]. Because a certain proportion of disease progression occurs within 6 months after radical CCRT, continuous administration of chemotherapy may help patients stay safe during this time period. In our study, we demonstrated that oral AC maintenance for >186 days (6 months) significantly improved OS and progression control. Owing to the accumulation of toxicity with no additional survival benefits after 12 months, the duration should not be infinitely prolonged.

Experts have concluded two approaches to develop AC in LA-NPC: refining more effective and better tolerated regimens, and investigating clinical and biological indices to identify patients with a high risk of relapse [45]. Because the pretreatment EBV DNA level was independent for OS in multivariate Cox regression analysis, we further explored its potential to stratify patient risks. In the high-risk subgroup, the mortality risk decreased to 1 at 194 days, and then slightly increased after 660 days. It suggests that patients with high tumour burden require longer oral AC maintenance after CCRT. But AC may only delay disease progression and death, not prevent them. Therefore, more aggressive therapeutic strategies should be considered for patients with pretreatment EBV DNA levels >4000 copies/mL.

This study had several limitations. First, this was a single-centre, retrospective study, which limits the generalisability of the results. Additionally, the sample size for the subgroup analysis was relatively small. Furthermore, the insufficient evidence for supporting the comparable efficacy of specific chemotherapeutic regimens should be considered. Finally, interruption or termination (tolerance of toxicities and decision of clinicians) of chemotherapy owing to various reasons might have affected the results.

In conclusion, the optimal duration of oral AC after CCRT was >186 days (6 months). The maintenance beyond 12 months may not bring additional benefits. For patients with high pretreatment EBV DNA levels, AC duration should be extended to 194 days. Further studies are warranted to validate these findings.

## Supplementary information

Supplemental Tables and Figures

## Data Availability

The datasets used and/or analysed during the current study available from the corresponding author on reasonable request.
