# Supplementary material for: Determining the optimal duration of oral adjuvant chemotherapy in locoregionally advanced nasopharyngeal carcinoma
Source: Br J Cancer. 2025 May 6;133(1):85–93. doi: 10.1038/s41416-025-03033-1 (PMC12238370; doi:10.1038/s41416-025-03033-1)
Supplement: Supplementary file 1 — Supplemental Tables and Figures [file 41416_2025_3033_MOESM1_ESM.pdf]

Supplemental Tables

eTable1. Inverse probability of treatment weighting-adjusted multivariate Cox regression analysis of secondary endpoints.

eTable2. Inverse probability of treatment weighting-adjusted univariate Cox regression analysis of overall survival.

eTable3. Baseline characteristics of the short and long duration groups before and after weighting in patients receiving induction chemotherapy.

eTable4. Multivariate Cox regression analysis of OS before and after weighting in patients receiving induction chemotherapy.

eTable5. Baseline characteristics of the short and long duration groups before and after weighting in patients not receiving induction chemotherapy.

eTable6. Multivariate Cox regression analysis of OS before and after weighting in patients not receiving induction chemotherapy.

Supplemental Figures

eFigure1. Restricted cubic spline modeling of the relationship between adjuvant chemotherapy duration and mortality risk.

eFigure2. Kaplan-Meier curves and the inverse probability of treatment weighting-adjusted Kaplan-Meier curves of overall survival for patients receiving induction chemotherapy (A, B) and patients not receiving induction chemotherapy (C, D).

Supplemental Tables

eTable1. Inverse probability of treatment weighting-adjusted multivariate Cox regression analysis of secondary endpoints.

Characteristic	HR	95%CI	p value
**PFS**			
Sex (Male vs Female)	1.41	0.83-2.39	.21
Age (≥45 vs <45)	1.06	0.63-1.77	.83
Family history of NPC (Yes vs None)	1.20	0.56-2.57	.64
T category[a] (T3-4 vs T1-2)	1.33	0.39-4.56	.65
N category[a] (N2-3 vs N0-1)	1.64	0.93-2.90	.09
Overall stage[a] (IVa vs III)	1.27	0.75-2.14	.37
Schemes (IC+CCRT+AC vs CCRT+AC)	1.28	0.76-2.16	.36
Pretreatment EBV DNA (>4000 vs ≤4000)	1.04	0.59-1.84	.89
Duration Group (Long vs Short duration)	0.51	0.32-0.83	.006
**LRRFS**			
Sex (Male vs Female)	0.98	0.52-1.86	.96
Age (≥45 vs <45)	0.90	0.46-1.75	.75
Family history of NPC (Yes vs None)	1.14	0.46-2.84	.77
T category[a] (T3-4 vs T1-2)	0.78	0.19-3.15	.73
N category[a] (N2-3 vs N0-1)	1.87	0.95-3.71	.07
Overall stage[a] (IVa vs III)	1.14	0.63-2.09	.66
Schemes (IC+CCRT+AC vs CCRT+AC)	1.14	0.58-2.23	.71
Pretreatment EBV DNA (>4000 vs ≤4000)	0.96	0.47-2.00	.92
Duration Group (Long vs Short duration)	0.47	0.26-0.86	.01
**DMFS**			
Sex (Male vs Female)	1.43	0.79-2.59	.24
Age (≥45 vs <45)	1.39	0.82-2.38	.22
Family history of NPC (Yes vs None)	1.49	0.60-3.68	.39
T category[a] (T3-4 vs T1-2)	3.01	0.84-10.77	.09
N category[a] (N2-3 vs N0-1)	1.73	0.89-3.38	.11
Overall stage[a] (IVa vs III)	1.81	0.95-3.46	.07
Schemes (IC+CCRT+AC vs CCRT+AC)	1.33	0.73-2.45	.36
Pretreatment EBV DNA (>4000 vs ≤4000)	1.74	0.96-3.14	.07
Duration Group (Long vs Short duration)	0.41	0.23-0.73	.002

[a]According to the eighth edition of UICC/AJCC staging system.

A Cox proportional hazards regression model was used to perform multivariate analysis. HRs and their 95% CIs were calculated for Sex (Male vs. Female); Age in years (≥45 vs. <45); Family history of NPC (Yes vs. No); T category (3–4 vs. 1–2); N category (2–3 vs. 0–1); Overall stage (IVa vs. III); Schemes (IC+CCRT+AC vs. CCRT+AC); Pretreatment EBV DNA (copies/mL) (>4000 vs. ≤4000); Duration group (Long duration vs. Short duration)

Abbreviations: HR = hazard ratio; CI = confidence interval; NPC = nasopharyngeal carcinoma; CCRT = concurrent chemoradiotherapy; AC = adjuvant chemotherapy; IC = induction chemotherapy; EBV = Epstein–Barr virus.

eTable2. Inverse probability of treatment weighting-adjusted univariate Cox regression analysis of Overall survival.

Characteristic	HR	95%CI	p value
Sex (Male vs Female)	0.91	0.40-2.11	.83
Age (≥45 vs <45)	1.03	0.46-2.31	.94
Family history of NPC (Yes vs None)	1.38	0.32-5.88	.66
T category[a] (T3-4 vs T1-2)	1.58	0.38-6.57	.53
N category[a] (N2-3 vs N0-1)	2.57	0.98-6.74	.05
Overall stage[a] (IVa vs III)	3.24	1.32-7.96	.01
Schemes (IC+CCRT+AC vs CCRT+AC)	2.08	0.91-4.73	.08
Pretreatment EBV DNA (>4000 vs ≤4000)	2.57	1.10-5.98	.03
Duration Group (Long vs Short duration)	0.23	0.10-0.55	<.001

[a]According to the eighth edition of UICC/AJCC staging system.

A Cox proportional hazards regression model was used to perform multivariate analysis. HRs and their 95% CIs were calculated for Sex (Male vs. Female); Age in years (≥45 vs. <45); Family history of NPC (Yes vs. No); T category (3–4 vs. 1–2); N category (2–3 vs. 0–1); Overall stage (IVa vs. III); Schemes (IC+CCRT+AC vs. CCRT+AC); Pretreatment EBV DNA (copies/mL) (>4000 vs. ≤4000); Duration group (Long duration vs. Short duration)

Abbreviations: HR = hazard ratio; CI = confidence interval; NPC = nasopharyngeal carcinoma; CCRT = concurrent chemoradiotherapy; AC = adjuvant chemotherapy; IC = induction chemotherapy; EBV = Epstein–Barr virus.

eTable3. Baseline characteristics of the short and long duration groups before and after weighting in patients receiving induction chemotherapy.

Characteristic	Unweighted, N%			Weighted, N%		
	Short duration n=61	Long duration n=94	p value	Short duration	Long duration	p value
Sex			.30			.79
Female	19(31)	37(39)		(34)	(36)	
Male	42(69)	57(61)		(66)	(64)	
Age			.03			.97
<45 years	26(43)	57(61)		(55)	(55)	
≥45 years	35(57)	37(39)		(45)	(45)	
Family history of NPC			.14			.92
No	59(97)	85(90)		(93)	(93)	
Yes	2(3)	9(10)		(7)	(7)	
T category*			.51			.62
T1-2	4(7)	9(10)		(6)	(8)	
T3-4	57(93)	85(90)		(94)	(92)	
N category*			.31			.98
N0-1	16(26)	32(34)		(32)	(32)	
N2-3	45(74)	62(66)		(68)	(68)	
Overall stage*			.049			.94
III	21(34)	19(20)		(24)	(23)	
IVa	40(66)	75(80)		(76)	(77)	
Pretreatment EBV DNA			.78			.99
≤4000 copies/mL	33(54)	53(56)		(55)	(55)	
>4000 copies/mL	28(46)	41(44)		(45)	(45)	

*According to the eighth edition of UICC/AJCC staging system.

The chi-squared test was used to calculate p value. All variables were transformed into categorical variables. Abbreviations: NPC = nasopharyngeal carcinoma; EBV = Epstein–Barr virus.

eTable4. Multivariate Cox regression analysis of OS before and after weighting in patients receiving induction chemotherapy.

Characteristic	Unweighted		Weighted	
	HR (95%CI)	p value	HR (95%CI)	p value
Sex	0.86 (0.32-2.31)	.76	0.87 (0.30-2.51)	.80
Female		Reference		
Male				
Age (years)	2.06 (0.74-5.76)	.17	1.83 (0.66-5.04)	.24
<45		Reference		
⩾45				
Family history of NPC	2.84 (0.31-26.33)	.36	4.78 (1.64-13.94)	.004
None		Reference		
Yes				
T category*	2.38 (0.29-19.31)	.42	2.73 (0.31-23.65)	.36
T1-2		Reference		
T3-4				
N category*	6.10 (0.77-48.24)	.09	6.90 (0.88-54.16)	.07
N0-1		Reference		
N2-3				
Overall stage*	2.07 (0.55-7.77)	.28	2.71 (0.77-9.54)	.12
III		Reference		
IVa				
Pretreatment EBV DNA (copies/mL)	2.27 (0.74-7.00)	.15	1.78 (0.63-4.96)	.27
⩽4000		Reference		
>4000				
Duration group	0.29 (0.10-0.81)	.02	0.27 (0.11-0.67)	.004
Short duration		Reference		
Long duration				

*According to the eighth edition of UICC/AJCC staging system.

A Cox proportional hazards regression model was used to perform multivariate analysis. HRs and their 95% CIs were calculated for Sex (Male vs. Female); Age in years (≥45 vs. <45); Family history of NPC (Yes vs. No); T category (3–4 vs. 1–2); N category (2–3 vs. 0–1); Overall stage (IVa vs. III); Pretreatment EBV DNA (copies/mL) (>4000 vs. ≤4000); Duration group (Long duration vs. Short duration)

Abbreviations: HR = hazard ratio; CI = confidence interval; NPC = nasopharyngeal carcinoma; EBV = Epstein–Barr virus.

eTable5. Baseline characteristics of the short and long duration groups before and after weighting in patients not receiving induction chemotherapy.

Characteristic	Unweighted, N%			Weighted, N%		
	Short duration n=67	Long duration n=34	p value	Short duration	Long duration	p value
Sex			.27			.98
Female	23(34)	8(24)		(30)	(30)	
Male	44(66)	26(76)		(70)	(70)	
Age			.44			.88
<45 years	27(40)	11(32)		(37)	(35)	
≥45 years	40(60)	23(68)		(63)	(65)	
Family history of NPC			.39			.98
No	61(91)	29(85)		(90)	(90)	
Yes	6(9)	5(15)		(10)	(10)	
T category*			.008			.96
T1-2	1(1)	5(15)		(6)	(6)	
T3-4	66(99)	29(85)		(94)	(94)	
N category*			.54			.99
N0-1	30(45)	13(38)		(43)	(43)	
N2-3	37(55)	21(62)		(57)	(57)	
Overall stage*			.53			.99
III	39(58)	22(65)		(57)	(57)	
IVa	28(42)	12(35)		(43)	(43)	
Pretreatment EBV DNA			.62			.77
≤4000 copies/mL	46(69)	25(74)		(69)	(72)	
>4000 copies/mL	21(31)	9(26)		(31)	(28)	

*According to the eighth edition of UICC/AJCC staging system.

The chi-squared test was used to calculate p value. All variables were transformed into categorical variables. Abbreviations: NPC = nasopharyngeal carcinoma; EBV = Epstein–Barr virus.

eTable6. Multivariate Cox regression analysis of OS before and after weighting in patients not receiving induction chemotherapy.

Characteristic	Unweighted		Weighted	
	HR (95%CI)	p value	HR (95%CI)	p value
Sex	2.18 (0.44-10.85)	.34	2.19 (0.43-11.06)	.34
Female		Reference		
Male				
Age (years)	1.04 (0.27-4.06)	.95	1.12 (0.29-4.27)	.87
<45		Reference		
⩾45				
Family history of NPC	0.99 (0.12-8.13)	.99	0.99 (0.16-5.96)	.99
None		Reference		
Yes				
T category*	0.56 (0.05-6.75)	.65	1.91 (0.09-39.15)	.67
T1-2		Reference		
T3-4				
N category*	0.61 (0.15-2.57)	.51	0.67 (0.21-2.16)	.50
N0-1		Reference		
N2-3				
Overall stage*	2.13 (0.51-8.88)	.30	1.77 (0.54-5.81)	.35
III		Reference		
IVa				
Pretreatment EBV DNA (copies/mL)	1.95 (0.50-7.52)	.33	1.97 (0.58-6.68)	.28
⩽4000		Reference		
>4000				
Duration group	0.15 (0.02-1.29)	.08	0.07 (0.009-0.59)	.01
Short duration		Reference		
Long duration				

*According to the eighth edition of UICC/AJCC staging system.

A Cox proportional hazards regression model was used to perform multivariate analysis. HRs and their 95% CIs were calculated for Sex (Male vs. Female); Age in years (≥45 vs. <45); Family history of NPC (Yes vs. No); T category (3–4 vs. 1–2); N category (2–3 vs. 0–1); Overall stage (IVa vs. III); Pretreatment EBV DNA (copies/mL) (>4000 vs. ≤4000); Duration group (Long duration vs. Short duration)

Abbreviations: HR = hazard ratio; CI = confidence interval; NPC = nasopharyngeal carcinoma; EBV = Epstein–Barr virus.

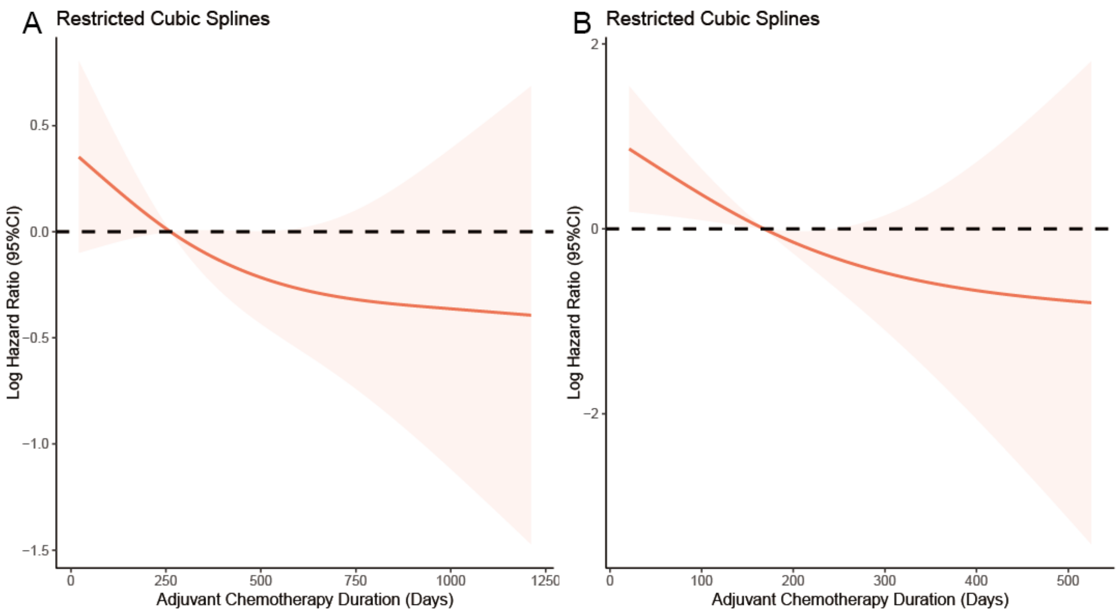

eFigure1. Restricted cubic spline modeling of the relationship between adjuvant chemotherapy duration and mortality risk.

For patients receiving induction chemotherapy (A) and not receiving induction chemotherapy (B), the hazard ratios (HR) derived from the multivariate Cox model, and the shaded areas represent the 95% confidence intervals (CIs) of the adjusted HRs.

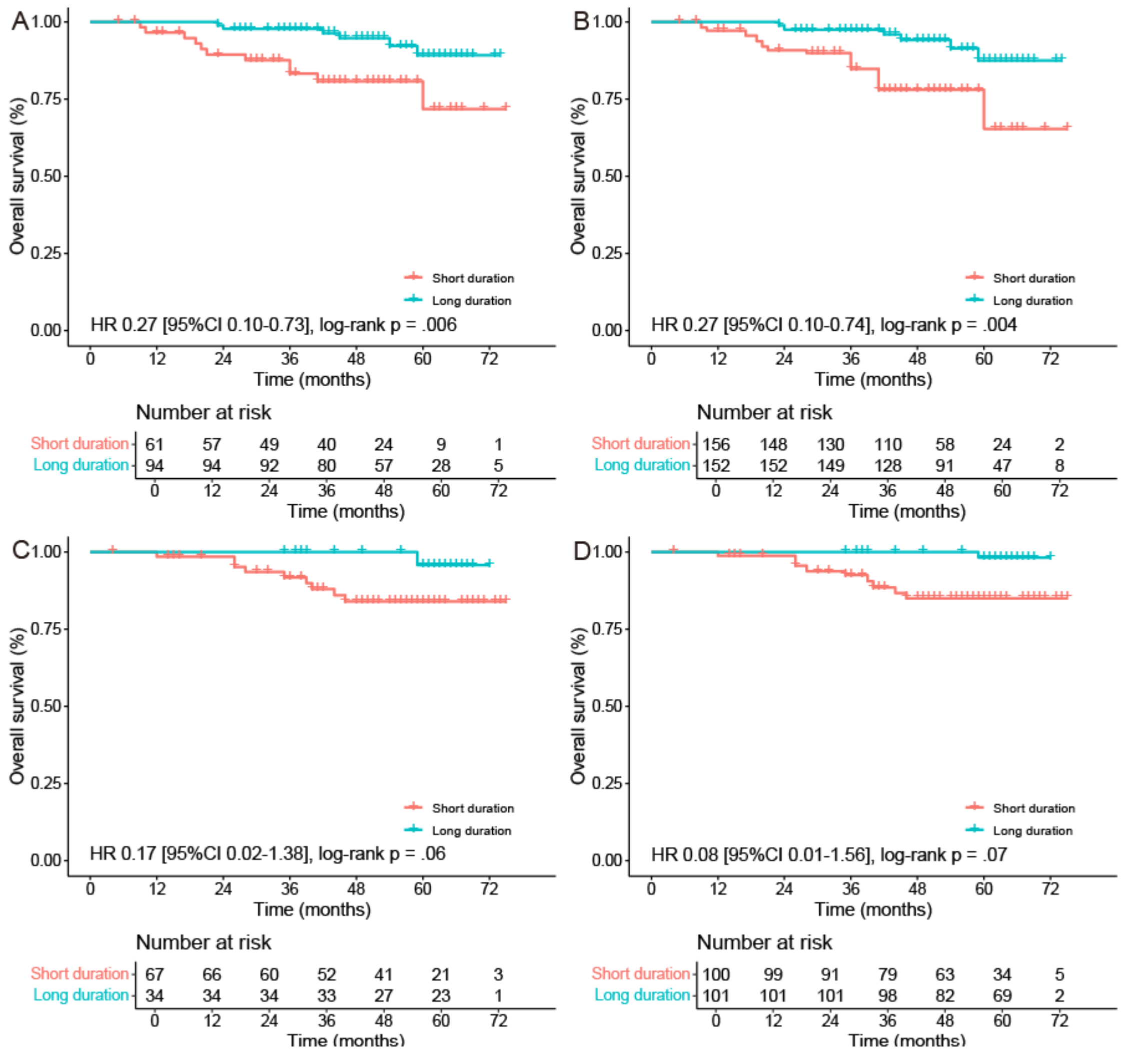

eFigure2. Kaplan-Meier curves and the inverse probability of treatment weighting-adjusted Kaplan-Meier curves of overall survival for patients receiving induction chemotherapy (A, B) and patients not receiving induction chemotherapy (C, D).

Univariate Cox proportional hazards model was used to calculate HRs and 95% CIs.
